# Ternary Solid Polymer Electrolytes at the Electrochemical Interface: A Computational Study

**DOI:** 10.1021/acs.macromol.3c02669

**Published:** 2024-04-29

**Authors:** Alejandro Rivera-Pousa, José Manuel Otero-Mato, Hadrian Montes-Campos, Trinidad Méndez-Morales, Diddo Diddens, Andreas Heuer, Luis Miguel Varela

**Affiliations:** † Grupo de Nanomateriais, Fotónica e Materia Branda, Departamento de Física de Partículas, Universidade de Santiago de Compostela, Campus Vida s/n, E-15782 Santiago de Compostela, Spain; ‡ Instituto de Materiais (iMATUS), Universidade de Santiago de Compostela, Avenida do Mestre Mateo 25, E-15706 Santiago de Compostela, Spain; § CIQUP, Institute of Molecular Sciences (IMS)—Departamento de Química e Bioquímica, Faculdade de Ciências da Universidade do Porto, Rua Campo Alegre, 4169-007 Porto, Portugal; ∥ Helmholtz-Institute Münster (HI MS), Ionics in Energy Storage, Forschungszentrum Jülich GmbH, Corrensstraße 46, 48149 Münster, Germany; ⊥ Institute of Physical Chemistry, University of Münster, Corrensstraße 28/30, 48149 Münster, Germany

## Abstract

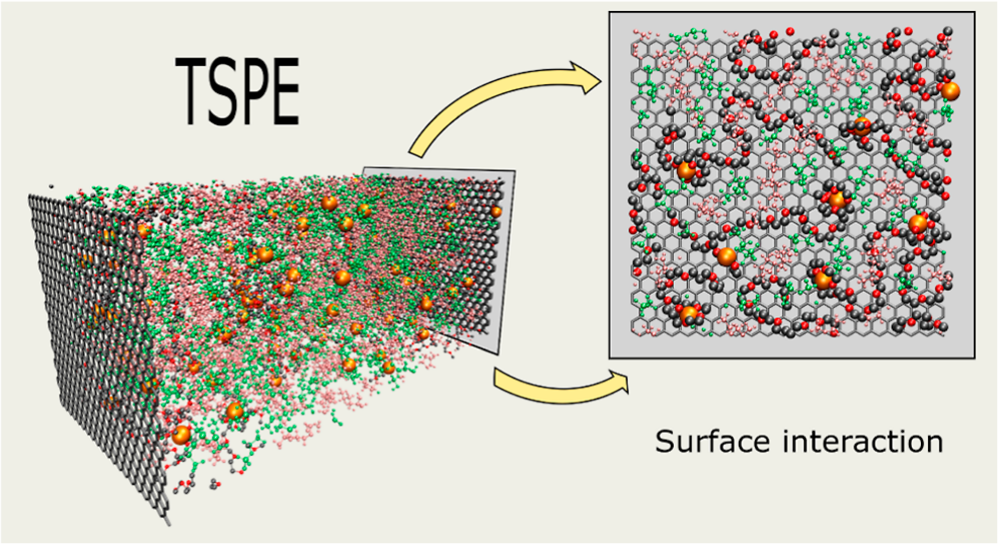

Polymer-based solid-like gel electrolytes have emerged as a promising alternative to improve battery performance. However, there is a scarcity of studies on the behavior of these media at the electrochemical interface. In this work, we report classical MD simulations of ternary polymer electrolytes composed of poly(ethylene oxide), a lithium salt [lithium bis(trifluoromethanesulfonyl)imide], and different ionic liquids [1-butyl-1-methylpyrrolidinium bis(trifluoromethanesulfonyl)imide and 1-ethyl-3-methylimidazolium bis(trifluoromethanesulfonyl)imide] confined between two charged and uncharged graphene-like surfaces. The molecular solvation of Li^+^ ions and their diffusion as well as the polymer conformational picture were characterized in terms of the radial distribution functions, coordination numbers, number density profiles, orientations, displacement variance, polymer radius of gyration, and polymer end-to-end distance. Our results show that the layering behavior of the ternary electrolyte in the interfacial region leads to a decrease of Li^+^ mobility in the direction perpendicular to the electrodes and high energy barriers that hinder lithium cations from coming into direct contact with the graphene-like surface. The nature of the ionic liquid and its concentration were found to influence the structural and dynamic properties at the electrode/electrolyte interface, the electrolyte with low amounts of the pyrrolidinium-based ionic liquid being that with the best performance since it favors the migration of Li^+^ cations toward the negative electrode when compared to the imidazolium-based one.

## Introduction

The current digital electronic revolution has gone hand in hand with the rapid growth of the number of portable electronic devices. Li-ion batteries are currently the dominant mobile power sources for these devices, since they have the highest energy per unit weight of the known energy storage systems.^1,2^ However, despite significant achievements in the optimization of Li-ion batteries, there are still notable challenges across cost, safety, and aging.^3,4^ For example, traditional lithium batteries use liquid electrolytes based on organic carbonates, which are highly flammable and volatile, and can lead to an inhomogeneous deposition of lithium in the form of dendrites.^5^

Solid polymer electrolytes (SPEs) have emerged as attractive candidates to solve these safety and life cycle issues. Their main advantages lie in having high flexibility, low cost, and enhanced thermal/electrochemical stabilities.^6,7^ Among all the options, poly(ethylene oxide) (PEO) has been demonstrated to possess an excellent salt-solvating ability. After being the first polymer electrolyte reported to dissolve Li ions in 1973,^8^ its potential use in lithium batteries was then explored in the pioneering works of Armand et al.^9−11^ Although PEO is one of the most studied polymers and most commonly used nowadays, PEO-based SPEs still suffer from lower ionic conductivity compared to conventional liquid electrolytes. While standard SPEs are prepared by dissolving a lithium salt in a polymer matrix, several strategies have been employed to enhance lithium-ion mobility. These approaches include, for instance, the use of lithium salts with noncoordinating anions such as bis(trifluoromethanesulfonyl)imide (TFSI),^12−18^ the dispersion of nanoparticles,^19−21^ or the addition of liquid plasticizers such as carbonates,^22,23^ among others.

Another feasible solution is to form ternary SPEs (TSPEs) by plasticizing the PEO-based SPE with an ionic liquid (IL). ILs are molten salts with melting points below 100 °C containing charge-balanced ions.^24,25^ Their properties can be modulated through different combinations of anions and cations, due to which they have been termed “designer solvents”. Their features, such as negligible volatility, high chemical and thermal stability, wide electrochemical window, and good solubility in various materials,^26,27^ make them promising green solvents for various applications including synthesis, catalysis, separation, lubrication, drug delivery, or energy storage.^28−30^ Although many works have considered ILs and their binary metal salt mixtures to be used as electrolytes in high-performance lithium-ion batteries,^31^ it has been observed that lithium salts not only usually exhibit poor solubility in ILs but also decrease the ionic conductivity of the binary mixtures upon increasing concentration.^32,33^ Also, these binary IL/salt electrolytes suffer from large anionic clusters, hindering lithium-ion transport. However, this inconvenient behavior is avoided when PEO is also present in the system, since it decouples lithium ions from its solvation shell and changes lithium transport to a PEO-dominated cationic mechanism.^34^ As a result, the addition of ILs to SPEs allows improving the conductivity of polymer electrolytes, increasing the number of charge carriers and accelerating polymer segmental dynamics, while guaranteeing high safety and good mechanical properties. Thus, it seems that TSPEs will play a fundamental role in next-generation energy storage technologies.

Mixtures of PEO, lithium salts, and pyrrolidinium-based ILs are undoubtedly the most investigated TSPE system, although short-chain imidazolium IL cations have also been considered.^35−41^ In addition to the increase in conductivity with IL addition, experiments on TSPEs have also reported aspects such as the effect of the temperature and their interfacial stability on lithium metal.^35−38,40,42−48^ For example, Passerini and co-workers observed that smaller anions such as bis(fluorosulfonyl)imide (FSI) increase electrical conductivity relative to TFSI at the expense of a lower thermal stability.^49^ They also highlighted the importance of the TSPE composition, since anions and polymers compete for the coordination with lithium cations.^50^

On the other hand, computational methods have been also widely employed over the last decades for the identification and understanding of promising TSPEs, since they allow the analysis of the systems at the molecular level and a straightforward comparison with the experimental measurements.^51,52^ In general, density functional theory (DFT) calculations have been employed to analyze either polymer decomposition on a metal surface^53,54^ or the local environment around lithium cations.^17^ Molecular dynamics (MD) simulations, which are much more abundant, have been mainly devoted to understand the structure of TSPEs and the mechanisms of lithium transport in them.^39,41,45,55−58^ For example, Diddens and Heuer and co-workers^59^ proved that the enhanced lithium diffusion in TPSEs including pyrrolidinium-based cations is due to the plasticization of the PEO backbone by the IL. Besides, they explained how the lithium-ion transport mechanism is modified when changing the composition of the TSPE: at high PEO/lithium ratios, the three transport mechanisms remain qualitatively the same as compared to binary SPEs [(i) diffusion of the cation along the PEO backbone, (ii) cooperative motion of the cation with the polymer segments, and (iii) cationic transfer between two polymer chains], whereas for low ratios, a fourth transport mechanism based on the coordination of lithium with TFSI anions becomes more relevant.^60^ Very recently, Paillard and co-workers^48^ combined MD simulations with experiments to suggest the use of coordinating anions like trifluoromethanesulfonyl-*N*-cyanoamide in TSPEs to accelerate lithium-ion transport by recoupling its dynamics from the polymer to the anion.

Moreover, while the bulk properties of binary and ternary SPEs have been extensively explored, their interfacial behavior at the proximity of an electrode is scarcely known. The interaction of the electrolyte with the electrode considerably modifies the properties of the liquids relative to their bulk values. However, the electrode/electrolyte interfaces are not well-understood yet. Thus, consideration of the structures and processes at the electrolyte/electrode interface is crucial for the optimization of energy storage devices. Concerning binary SPEs,^61,62^ it was observed by Thum et al.^63^ using MD simulations that the presence of a surface does not affect the parallel transport mechanism, but it substantially reduces the lithium-ion diffusion in the direction perpendicular to the interface. To the best of our knowledge, only one work analyzing the interfacial properties of TSPEs at a Li-metal surface has been reported up until now.^64^ In that case, several combinations of IL cations and anions were tested to tune the structural and dynamic properties at the surface/electrolyte interface, and 1-ethyl-3-methylimidazolium 1,2,3-triazolide ([EMIM][123Triaz]) was observed to show the best performance.

In this work, with the aim of getting a better understanding of the electrode/electrolyte interface, we performed MD simulations of ternary PEO-based electrolytes to analyze their bulk behavior and the interfacial properties at a graphene-like surface (both under charged and uncharged conditions). The studied ternary system is composed of the polymer PEO; a lithium-based salt, [Li][TFSI]; and an IL. Two different ILs were considered, both sharing the anion with the salt species: [EMIM][TFSI] and [PYR_14_][TFSI], and for the imidazolium-based TSPE, two concentrations were considered. Thus, in this contribution, the following questions will be addressed:

- How does the presence of a graphene-like surface influence the structure of the TSPE mixtures in the interfacial region? Moreover, how does the conformational redistribution depend on the surface charge?
- What is the influence of the IL on the interaction between the lithium cation and the polymer?
- Does the concentration of the TSPE or the structure of the IL cation have any impact on the interfacial properties? Which mixture shows the best potential as battery electrolytes?

Our findings are presented as follows: In the next section, details of the simulation setup are given. Then, the results are discussed in the section and, finally, the main conclusions are summarized.

## Simulation Details

The reported simulations were carried out using Gromacs 2019.5 software.^65,66^ The OPLS-AA^67^ force field was used to describe the molecular interactions of PEO, Li^+^, and EMIM^+^. PYR_14_^+^ and TFSI^–^ were parameterized according to the CL&P force field,^68−70^ which is compatible with OPLS-AA. On the basis of a mean-field approximation, a charge scaling of 0.8 was employed to take into account polarization effects, thus obtaining a better agreement with experiments compared to a full charge model. This methodology has been previously used to investigate SPEs.^18,55^

The polymeric PEO chains were composed of 19 monomers, that is, 20 ether oxygens. Already coiled structures were chosen as the dynamics of the system is very slow. The base ratio between the different species was chosen as 20:2:6, i.e., for each 20 PEO oxygen ethers (1 chain), 2 salt molecules and 6 IL molecules are present. In order to study the influence of the IL concentration on the properties of the system, the [EMIM][TFSI]-based setups were also analyzed at an oxygen(PEO)/salt/IL ratio of 20:2:20. The initial configuration was obtained following the algorithm described in ref (56) that allows encapsulation of long molecules, such as polymers, in periodic simulation boxes. This method takes advantage of the software package Packmol.^71^ Bulk simulations of all of these mixtures were carried out using 25 polymer chains and the respective amounts of lithium salt and IL.

The simulation path followed was the one thoroughly described in ref (63). It starts with two energy minimizations, using first a steepest descent followed by a conjugated gradients algorithm. Then, two implicit graphene-like walls were inserted in the *z* dimension at the ends of the simulation box using the Gromacs tool. These layers interact with the electrolyte as a surface-integrated 10-4 Lennard-Jones potential. The lateral box dimensions were fixed throughout the whole preparation and simulation of the systems in order to afterward adjust to the graphene-like sheet size, ensuring correct periodicity of the C–C bonds. For this purpose, the *x* and *y* dimensions were set to 4.9118 and 4.6798 nm, respectively. As the lateral size of the simulation system cannot change, the *z* dimension will have to be accommodated to recover the correct bulk density. The exact number of molecules of each species can be seen in Table 1 and was chosen as to obtain approximately 10 nm between graphene-like walls, which was deemed sufficient to recover bulk conditions in the center of the simulation box.

**Table 1:** Number of Molecules Contained in the Simulation Box for the Different Systems

system	concentration	*n* PEO	*n* [Li][TFSI]	*n* IL
[Pyr_14_][TFSI]	20:2:6	45	90	270
[EMIM][TFSI]	20:2:6	51	102	306
[EMIM][TFSI]	20:2:20	21	42	420

Periodic boundary conditions are applied in the *x* and *y* dimensions. After energy minimization, 3 ns 273 K *NVT* and 3 ns 423 K *NVT* runs were done as a first attempt to relax unfavorable configurations. The temperature was maintained using a Berendsen thermostat.^72^ Then, a series of *NPT* stabilizations with semi-isotropic Berendsen pressure coupling^72^ allowed the *z* dimension of the box to adapt to reach the right density. An initial NPT run of 3 ns was followed by an annealing at 623 K for 100 ns, eventually followed by a temperature decrease to the initial 423 K and another 100 ns of NPT relaxation.

After this procedure, we removed the implicit walls and inserted the real graphene-like surfaces. The carbon atom selected was opls147. The graphene-like surfaces were built using the carbon nanostructure utility of the software package VMD.^73^ These graphene-like walls will remain fixed throughout the simulation procedure; therefore, the box dimensions will not further change. The systems were simulated both with no charge on the carbon atoms of the graphene-like walls as well as with a charge density of σ = ±1*e*/nm^2^, which results in a charge per carbon atom of ±0.0131*e*. The atomic charge is kept constant throughout the simulation, and no image charge effects are considered. Coulomb interactions were taken into account using the smooth particle mesh Ewald (PME) method^74,75^ with the Yeh–Berkowitz correction.^76^ Although constant potential conditions would result in a more realistic simulation environment, it is a technique with a much larger computational cost. The main difference in predictions from both techniques comes in the dynamics of the formation of the electric double layer,^77^ but the main focus of this work is the structuring of the mixture. Furthermore, it has been previously shown in the literature that for space widths larger than 1.4 nm, the constant charge method produces reliable counterion concentration results.^78^ Adding the extra computational cost to the already long simulations needed for systems with slow dynamics, we deemed appropriate the use of the constant charge technique in this study.

After a final energy minimization was carried out, a 600 ns *NVT* run applying a Nosé–Hoover thermostat^79^ was used as the production run, where we discarded the results from the first 100 ns since they were considered as further stabilization of the system, as can be seen in Figure S1 of the Supporting Information. For the systems with charged graphene-like surfaces, the configuration after the first 100 ns of the neutral system was selected as a starting point. The graphene-like surface at *z* = 0 nm is always positively charged, and the other surface is always negatively charged. These systems were simulated in a manner similar to that for the neutral ones, discarding the first 100 ns of the 600 ns *NVT* production run. Even though such long simulation times are computationally expensive, they are necessary to allow for slow dynamics of these systems. Simulations of equivalent systems but without the polymer molecules were carried out for further comparison.

## Results and Discussion

### Influence of the Presence of a Graphene-like Surface

**Figure 1:**
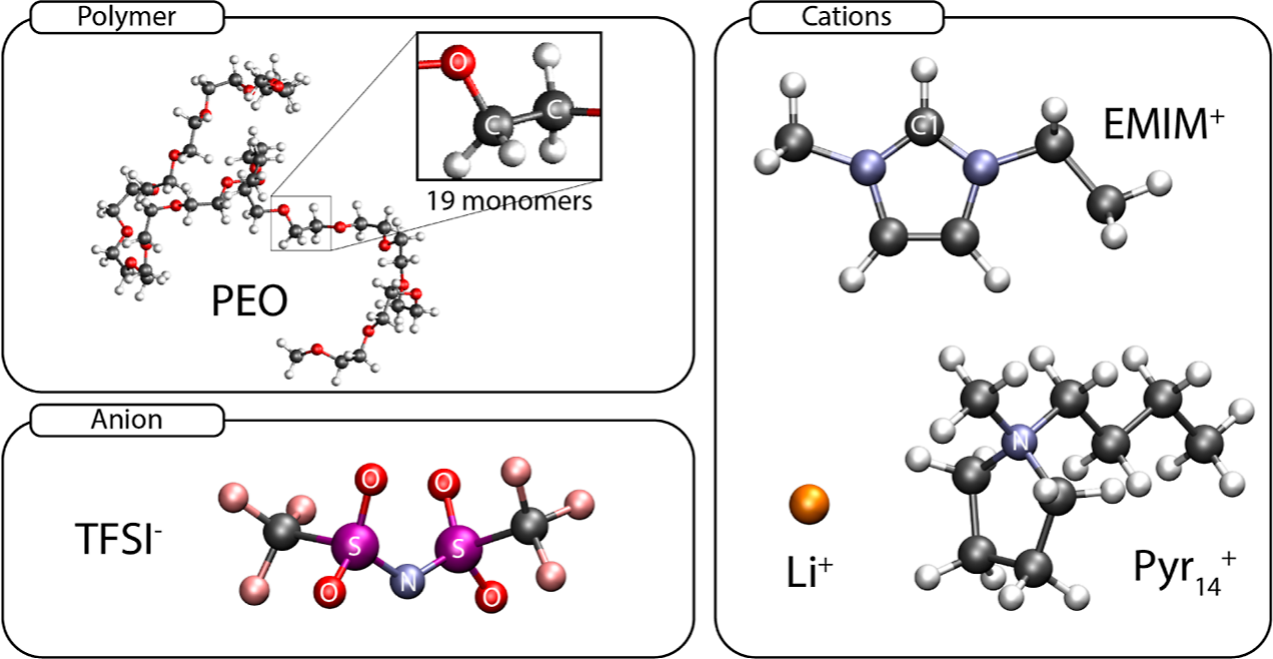
3D models of the species present in the systems. Oxygen, carbon, hydrogen, nitrogen, sulfur, fluorine, and lithium atoms are represented in red, black, white, blue, purple, pink, and orange, respectively. Relevant sites employed for the calculations in each species are explicitly labeled.

**Figure 2:**
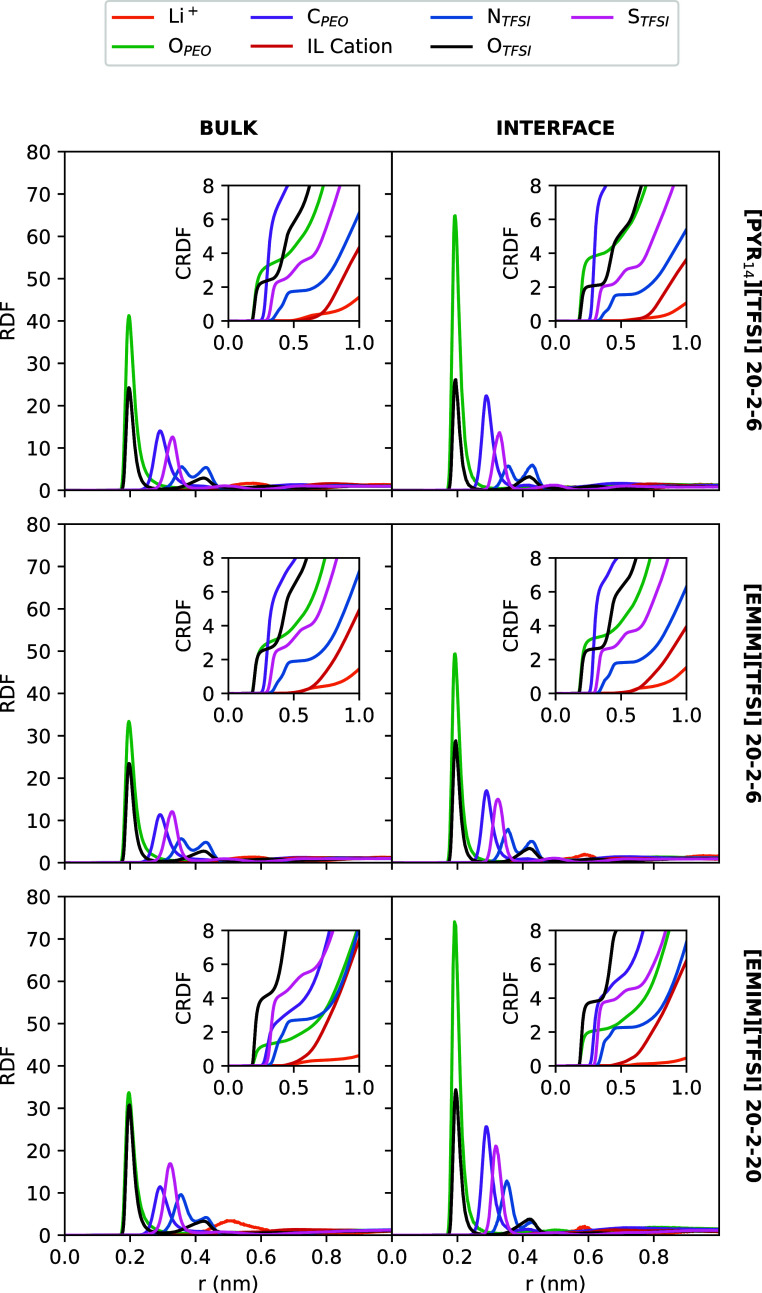
RDFs between Li^+^ and several atoms of the different molecules in bulk mixtures (left) and in the interfacial region in systems between neutral graphene-like walls (right). The insets show the corresponding cumulative RDF. The representative atoms for TFSI^–^ and PEO are specified in the legend, whereas C1 and N were chosen for EMIM^+^ and Pyr_14_^+^, respectively.

In order to analyze the structural properties of the TSPEs, we computed the radial distribution functions (RDFs) of the different species of the mixture around a Li^+^ cation. In this section, these functions were calculated both in bulk and in the interfacial region of the slab model (∼2 nm from the neutral graphene-like surface), as seen in Figure 2. For that purpose, we selected relevant sites of the molecular components (shown in Figure 1) to achieve a comprehensive view of the structural arrangement of the system. Thus, for the IL cation, C1 and N atoms were chosen for the imidazolium and pyrrolidinium rings, respectively, whereas those selected for the other species are specified in Figure 2. There we can clearly observe that Li^+^ prioritizes the oxygen atoms from the molecular components, both PEO and TFSI^–^, thus evidencing a mixed coordination in agreement with previous computational and experimental works on TSPEs.^38,41^ Although the Li^+^ environment is similar in both configurations, the height of those peaks increases in the presence of the graphene-like surface, especially the coordinations that involve the polymer molecule. This is indicative of the ternary mixtures becoming more ordered when confined due to organization of the molecules in the interfacial region. Other sites of the molecules appear as the following closest atoms as a consequence. Specifically, the two peaks for the TFSI^–^ nitrogen interaction confirm that Li^+^ and TFSI^–^ coordinate in two different conformations: monodentate and bidentate, which are yielded by the well-known *cis* and *trans* conformations of TFSI^–^ and are typical of this kind of aggregate.^80,81^ It is interesting to note that concentration seems to play a similar role both in the bulk and at the interface since more IL concentration leads to the bidentate coordination being more abundant. However, no remarkable effects on the height of the peaks concerning the TFSI^–^ oxygen atoms are induced by increasing the amount of IL.

**Figure 3:**
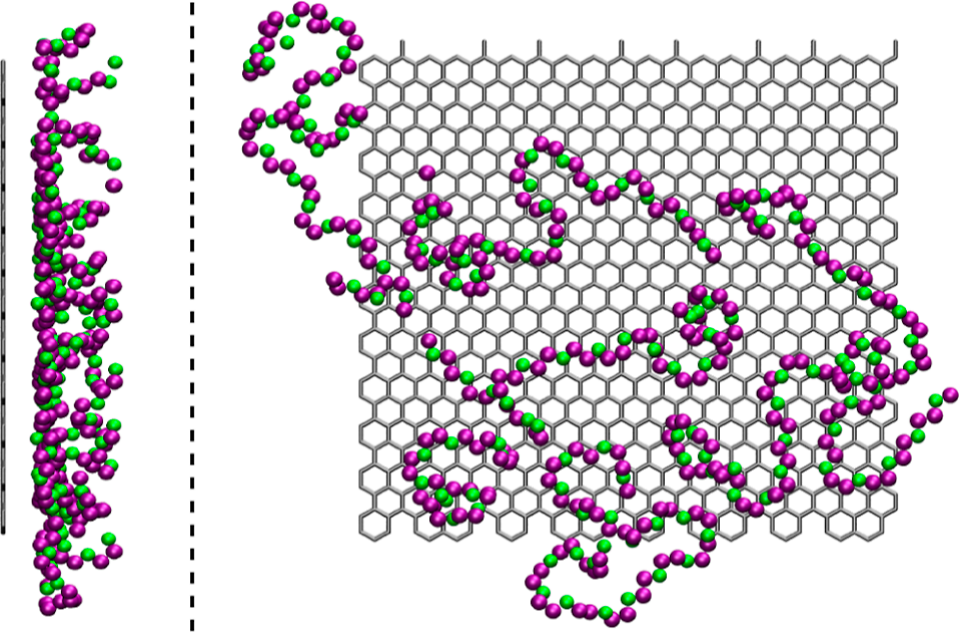
Snapshots in the *YZ* plane (left) and *XY* plane (right) of the polymer chains closer than 1 nm to a neutral electrode in mixtures with [Pyr_14_][TFSI]. Oxygen and carbon atoms are represented in green and purple, respectively.

The cumulative RDFs (CRDFs), i.e., the average number of particles within a distance *r* around a central molecule, also provide us with information about the spatial distribution. The results for Li^+^ solvation are included in the insets of Figure 2. As we can see, in bulk simulations, Li^+^ coordinates in its first solvation shell with around 3 oxygen atoms of PEO and 3 oxygen atoms of TFSI^–^ (depending on the system), giving an average coordination number of 6, which is typical for this kind of electrolyte system.^41^ An increase in IL concentration leads to the coordination with TFSI^–^ taking over and the coordination with the polymer exhibiting the inverse trend, although keeping the total amount of 6 oxygens being coordinated with a Li^+^ cation. This change in the coordination environment of lithium cations through a weakening of the so-called “breathing mode” (the association between Li^+^ and PEO) is mainly due to the decrease in the polymer fraction as the IL content increases. This structural picture is slightly modified when the system is under confinement. At the neutral interface, the total number of oxygen atoms coordinating a Li^+^ cation is maintained, with the “breathing mode” being favored when compared to the bulk. Thus, the neutral graphene-like surface gets Li^+^ preferentially coordinated to the PEO oxygens. The opposite behavior, that is, an increase in the number of TFSI^–^ anions in the lithium-ion solvation shell when going from the bulk to the interface, was previously reported when introducing a Li-metal surface.^62,64^ This discrepancy is an effect of the nature of the electrode on its interaction with the polymer. That is, due to the well-known hydrophobicity of graphene,^82,83^ this carbon surface shows stronger interactions with the PEO carbon atoms than the Li-metal surface, resulting in a larger number of polymers in the interfacial region that are available for coordinating a lithium ion, as it will be shown by the calculation of the number density profiles included in Figure 5. In fact, this can be clearly appreciated in the snapshot included in Figure 3 (right), where the polymers in the proximity of an uncharged graphene-like sheet are shown to be arranged parallel to the surface, thus maximizing their interaction with the neutral electrode. This will be also confirmed later with the analysis of the orientations.

### Influence of the Charge of the Graphene-like Surface

**Figure 4:**
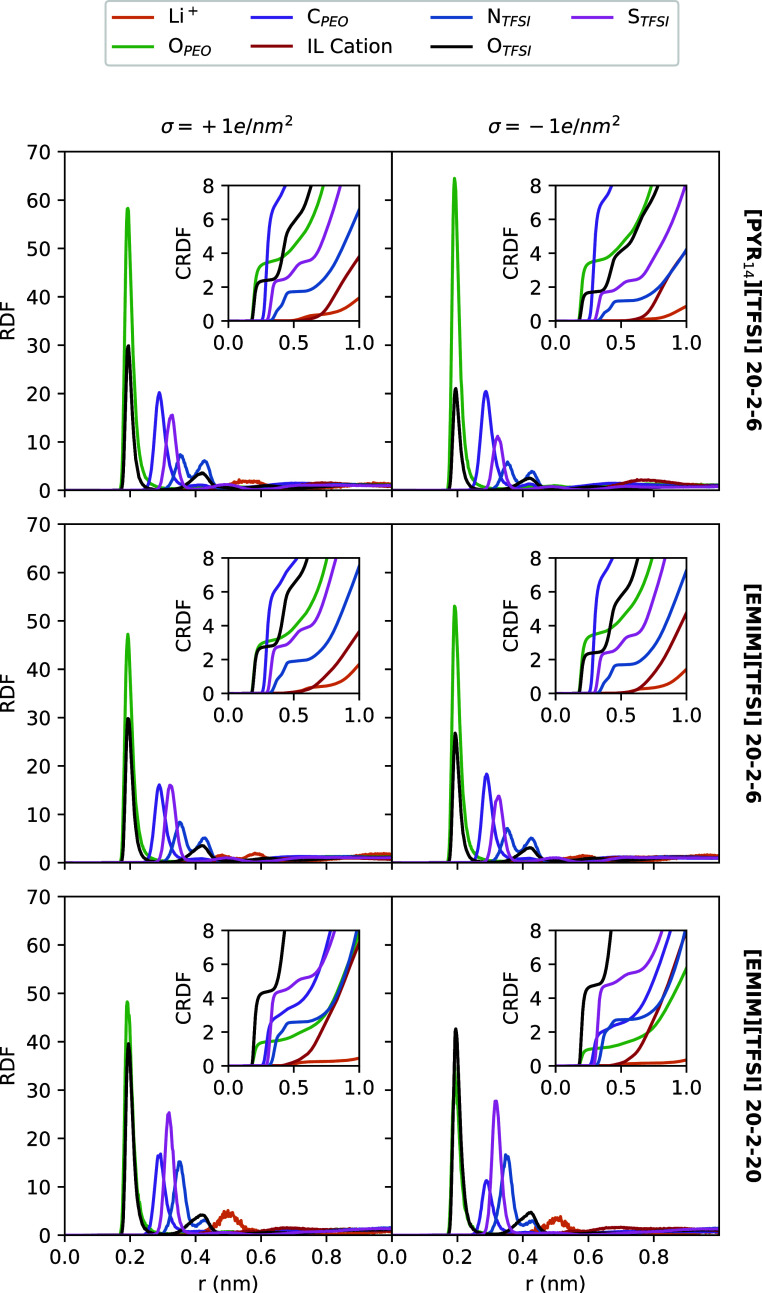
RDFs between Li^+^ and several atoms of the different molecules in the region near the positively (left) and negatively (right) charged graphene-like surfaces, respectively. The insets show the corresponding cumulative RDF. The representative atoms for TFSI^–^ and PEO are specified in the legend, whereas C1 and N were chosen for EMIM^+^ and Pyr_14_^+^, respectively.

RDFs and CRDFs in the interfacial regions with charged graphene-like electrodes were calculated with the aim of analyzing the impact of charging the surface. The corresponding plots are shown in Figure 4.

At surface charges of σ = +1*e*/nm^2^, the interaction between an oxygen of the polymer and a Li^+^ cation is always slightly lower than that at neutral interfaces, whereas that with an oxygen from the TFSI^–^ anion is greater. In fact, it can be seen that the interaction between lithium and the anion increases when increasing the amount of IL in the ternary mixture due to the presence of a greater number of TFSI^–^ anions. Concerning the coordination numbers, in the first solvation shell of lithium ions, there are always around 2–3 oxygens of PEO and 2–4 oxygens of TFSI^–^ anions. Since the anions are expected to be located in the neighborhood of the positively charged surface, this could be indicative of Li^+^ showing correlated ion motion with the anions in negatively charged Li-containing clusters. This will be explored later with an analysis of the number density profiles.

On the other hand, at surface charges of σ = −1*e*/nm^2^ and low amounts of IL, the interaction between an oxygen from PEO and a solvated Li^+^ ion is stronger than at positively charged surfaces, but it decreases when increasing the concentration. Regarding the interaction of Li^+^ with the oxygens of TFSI^–^ anions, the height of the peaks of the RDF is comparable to that in neutral interfaces, with a tendency to increase with IL concentration. Lithium coordination numbers with the polymer are 3 (except for the most concentrated mixture), as observed in the vicinity of the positive electrode, whereas those with the anion show an increase with the amount of IL, probably due to a greater number of TFSI^–^ anions being available for solvating the same number of lithium cations.

**Figure 5:**
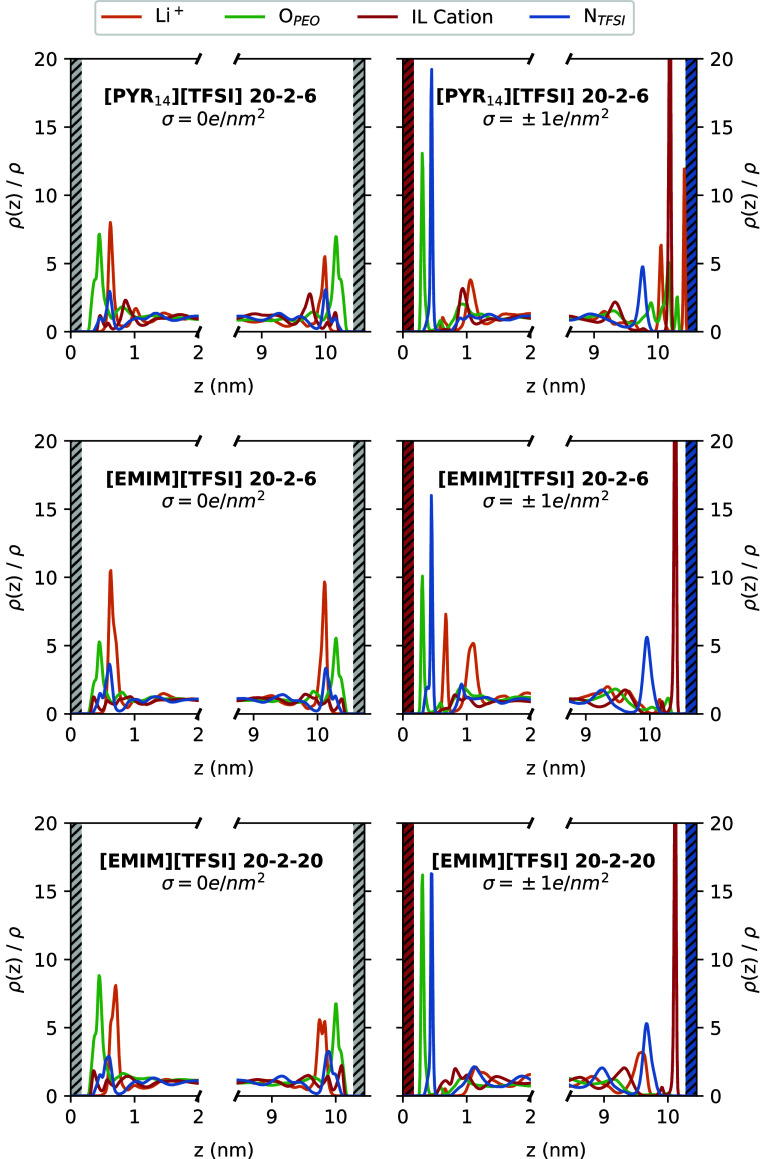
Density profiles in the *z* direction, normalized to the bulk density, for several atoms of the different molecules in systems confined between neutral graphene-like surfaces (left) and charged graphene-like surfaces (right). The representative atoms for TFSI^–^ and PEO are specified in the legend, whereas C1 and N were chosen for EMIM^+^ and Pyr_14_^+^, respectively.

As it is well-known, the interface leads to a layering of highly concentrated ionic electrolytes due to the interactions between the electrolyte and the surface.^84−86^ To analyze this effect and shed more light on the microscopic structure of these systems, we also computed the density profiles along the *z*-axis, which are displayed in Figure 5. The first characteristic that is visible there is that some degree of layering in the interfacial region takes place also at neutral surfaces, which is associated with the translation symmetry breaking, but the effect is much more pronounced when charging the graphene-like sheet. In both cases, bulk density is recovered after approximately 2 nm from the carbon surface.

**Figure 6:**
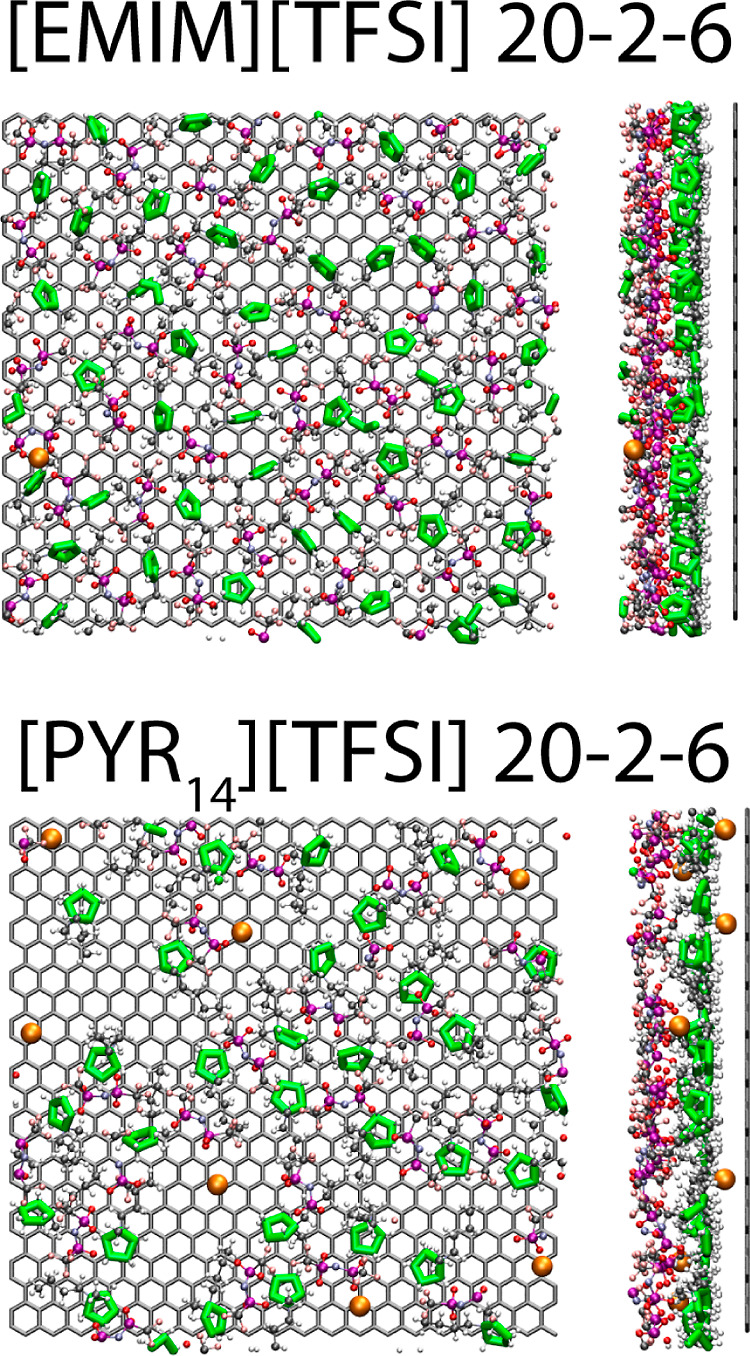
Snapshots in the *XY* plane (left) and *YZ* plane (right) of the molecules closer than 1 nm to the negative electrode in the [EMIM][TFSI] (top) and [Pyr_14_][TFSI] (bottom) 20–2–6 systems. Lithium atoms, in orange, and cationic rings, in green, are artificially enlarged for better visual clarity.

On the other hand, as can be seen in Figure 5 (left) for uncharged graphene-like surfaces, Li^+^ atoms are never found in the regions adjacent to the walls, regardless of the IL employed and the concentration of the TSPE. Instead, they are located sharing the second layer with TFSI^–^ anions. In turn, the first layer is mainly occupied by PEO molecules and a small amount of IL ions. This behavior has been also observed at the interface between SPEs and a Li-metal surface, and it is indicative of a preference of the surface to interact with the electron-rich compounds.^62,64^ Li^+^ cations are more likely to interact with the electrolyte molecules rather than with the surface atoms. On the other hand, it is also shown in Figure 5 (right) that charging the graphene-like electrode leads to the spontaneous accumulation of counterions and the depletion of co-ions near the charged surface, as expected. Interestingly, an inner PEO layer emerges directly attached to the positively charged carbon surface at around 0.3 nm, followed by a dense layer of anions at secondary positions. Concerning the negatively charged surface, a dense layer of IL cations is always formed at approximately 0.4 nm from the graphene-like wall, but some differences can be seen between the different systems. First, for the lowest IL concentrations (20–2–6), lithium cations are able to directly approach the electrode together with some PEOs, which are split in several layers, with the pyrrolidinium-based TSPE but not with the imidazolium-based one. Apparently, with [Pyr_14_][TFSI], Li^+^ is not able to desolvate from the polymer, but these positively charged aggregates are indeed capable of reaching the negative electrode. This can be also observed in the snapshots of Figure 6, where the 1 nm-wide regions from the negative surface are represented for these two mixtures. However, when low amounts of [EMIM][TFSI] are present in the electrolyte, Li^+^ seems to keep a strong coordination with TFSI^–^ anions, as shown by the emerging lithium-ion populations at around 0.7 and 1.1 nm from the positively charged graphene-like sheet. This is probably due to the stronger interaction of TFSI^–^ anions with Pyr_14_^+^ than with EMIM^+^ that diminishes the degree of anion clustering with Li^+^. This can be observed in Figure S2 of the Supporting Information, where we have plotted the RDFs between the IL ions for binary mixtures of [EMIM][TFSI] with LiTFSI and [Pyr_14_][TFSI] with the same salt, both with a ratio of 6:2, which corresponds to the lowest IL concentration in the TSPEs.

**Figure 7:**
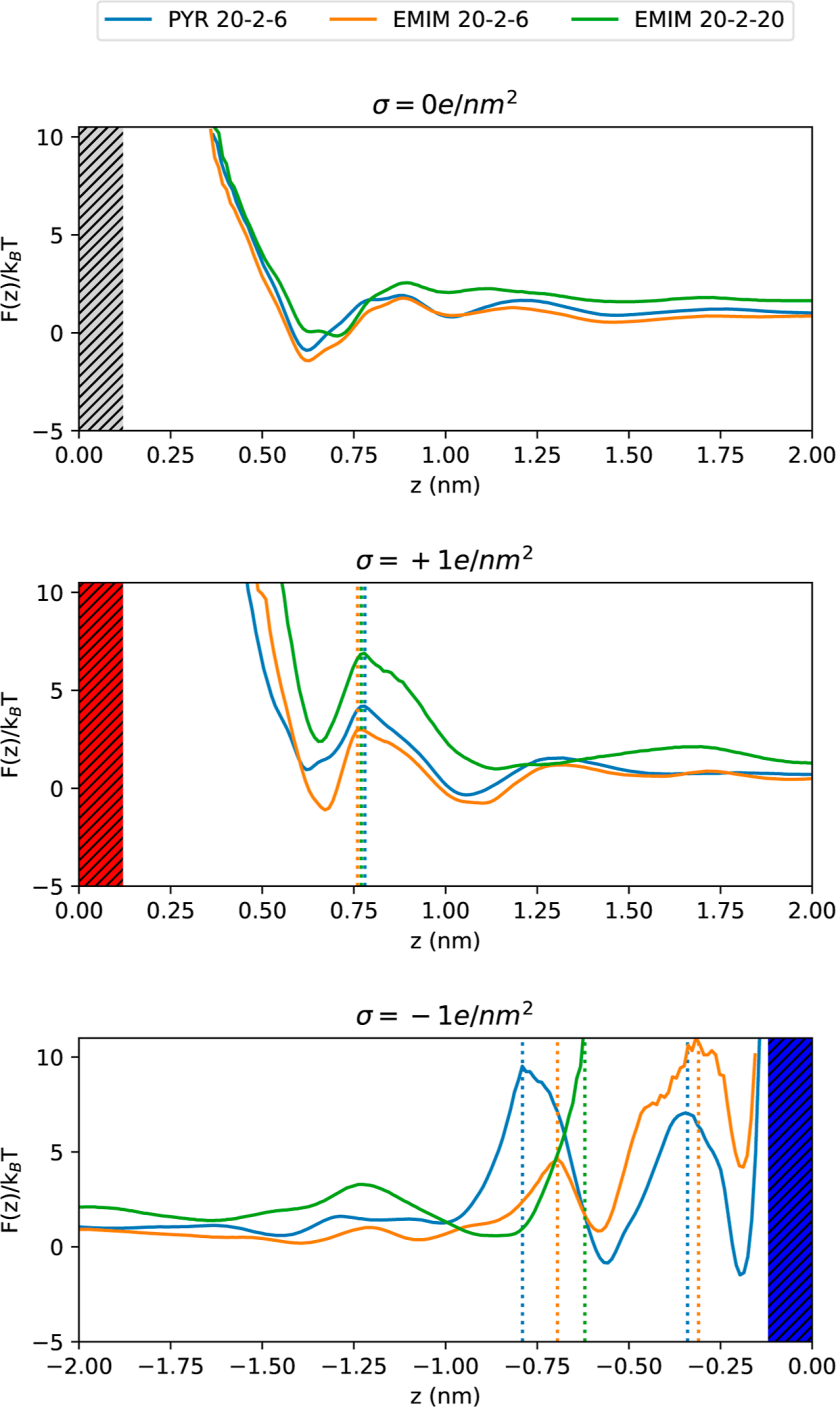
Free-energy profiles for lithium ions approaching the graphene-like surfaces with neutral (top), positive (center), and negative (bottom) charge densities for the investigated TSPEs. Dotted lines show the positions of the barrier for all the concentrations: PYR 20–2–6 (blue), EMIM 20–2–6 (orange), and EMIM 20–2–20 (green). In all cases, the corresponding electrode is located at *z* = 0.0 nm.

Focusing now on the PEO/[Li][TFSI]/[EMIM][TFSI] electrolyte between charged surfaces, it can be observed in Figure 5 that Li^+^ cations are not able to cross the dense layer of EMIM^+^ and approach the negatively charged carbon surface for any of the tested concentrations. Thus, we try to get a deeper understanding about the movement of Li^+^ cations calculating the free-energy profiles of transferring a Li^+^ from the bulk to the graphene-like surface as
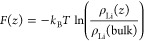
1where *k*_B_ is the Boltzmann constant. The free-energy profiles obtained for uncharged, positively charged, and negatively charged surfaces are included in Figure 7, where we can observe the oscillations due to the layering behavior of the TSPE in the proximity of the wall. At uncharged surfaces, for all the systems, there is an easily accessible first minimum at 0.6 nm followed by a second one at 1 nm. This shows that a given lithium is slightly stabilized at uncharged interfaces, with a larger lifetime in the minimum at 0.6 nm. However, it should be kept in mind that in the neutral slab configuration, there is an inner layer composed mainly of PEO and some IL that impedes Li^+^ from coming into direct contact with the surface.

In the case of positively charged interfaces, the opposite behavior can be observed for most systems. That is, the Li^+^ cation has a preference to place at distances larger than 1 nm rather than in the first layer, the only exception being the electrolyte with the lowest amount of [EMIM][TFSI]. In that mixture, Li^+^ faces the lowest energy barrier to jump into the first layer. Once it has entered it, the Li^+^ ion will stay there for some extended waiting time. This behavior arises from the strong interaction between Li^+^ and the first layer of TFSI^–^ anions at the positive electrode, as we have seen in Figure 5. At the negative electrode, the height of the free-energy barriers increases remarkably in comparison to the previous situations due to a depletion of Li^+^ cations in some regions, as shown in Figure 5. Now, free-energy barriers are much higher than thermal energy, so the diffusion process toward the negative electrode can be regarded as a rare event, with the [Pyr_14_][TFSI]-based TSPE showing the highest probability of Li^+^ approaching the graphene-like surface. However, if a rare transition from the second layer at 0.6 nm to the innermost one at 0.2 nm takes place, which has been observed to happen 11 times during the whole trajectory, then Li^+^ cations are accommodated in a stable position with high energy barriers for escaping from the negative electrode, since for that to happen, they must overcome the electrostatic attraction of the electrode.^63^ In fact, our simulations show that once Li^+^ cations reach the negative electrode, they remain at the innermost layer (on average) during 24.15 and 3.00 ns for PYR 20–2–6 and EMIM 20–2–6, respectively.

Another relevant aspect of the TSPE studies in this work is the particular orientation that the long polymeric chains adopt near the interfaces. As it has been shown in previous studies,^59^ the polymer chains allow three new possible channels of Li^+^ diffusion in these mixtures: (i) motion along with the chain, (ii) hopping between chain sites, and (iii) hopping between chains. As a result, the orientation of these chains could play an important role in the diffusion of Li^+^ cations toward the electrodes. To analyze this feature, we considered the orientations of the different species in the system by computing the probability density distribution of the cosine of the relative angle between a vector normal to the surfaces pointing to the bulk and a characteristic molecular vector. For the IL cation, the vector is normal to the ring and has the geometrical center of the ring as the application point. For the polymer, a subdivision of the chains allows better identification of the correct orientation of the chains. Thus, we decided to take multiple vectors between consecutive oxygen sites of PEO, that is, going from one monomer to the next one in a random direction, with the application point of the vector being placed halfway between the two oxygens. For both species, the orientations were calculated only for those molecules whose application point of the characteristic vector lies within the first 2 nm from the charged and uncharged electrodes.

**Figure 8:**
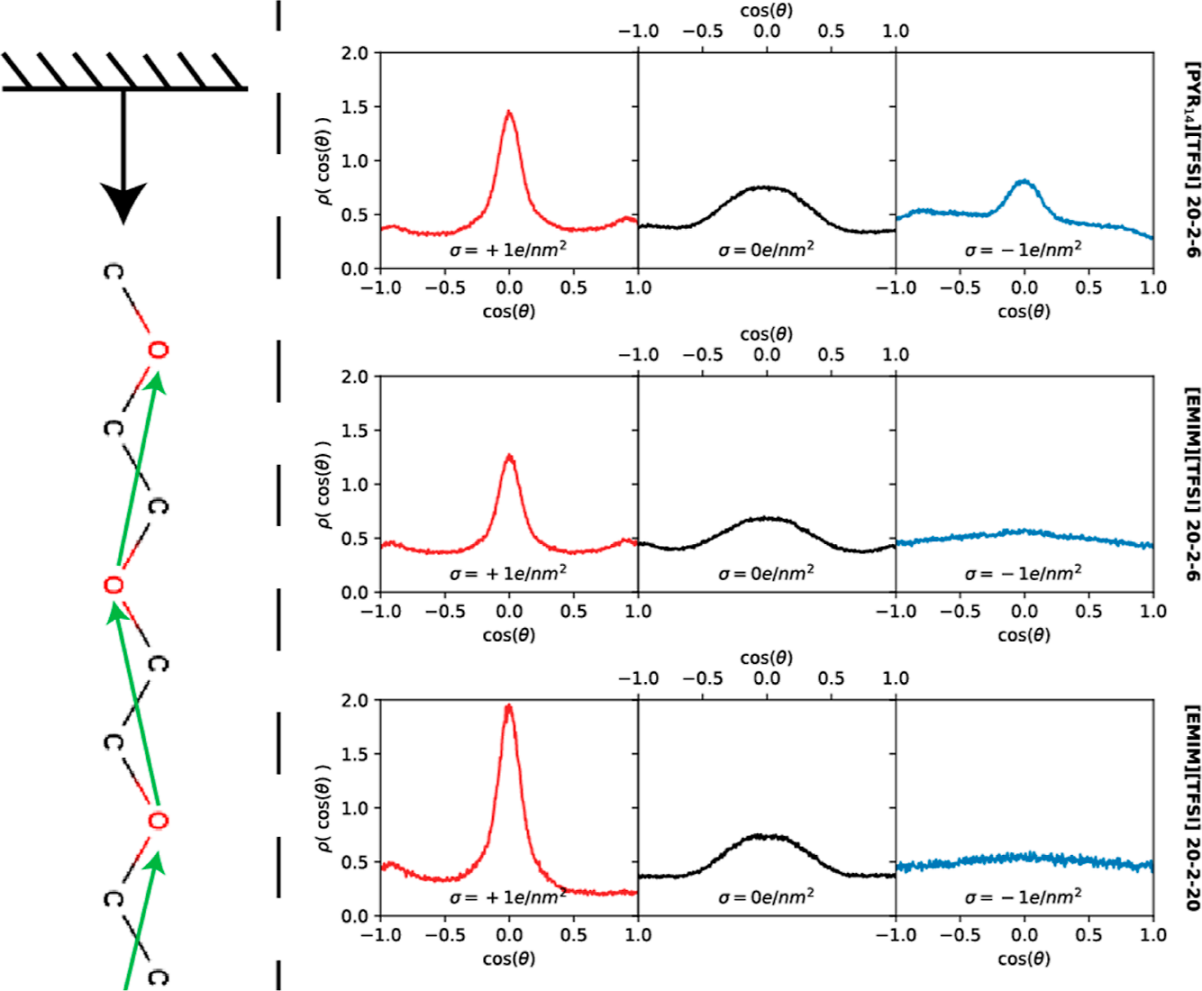
Marginal probability density distribution of polymer orientation relative to the vector normal to the graphene-like surface in the interfacial region (up to 2 nm). A scheme depicting the characteristic vectors defined for the polymer is shown on the left.

The orientations of the interfacial PEO molecules are plotted in Figure 8. There we can observe higher populations of polymers in the parallel orientations close to the graphene-like surfaces for most of the considered systems, thus trying to maximize their interaction with the carbon atoms of the surface, as was previously mentioned in the analysis of the effect of confining the SPE. This arrangement is more marked in the vicinity of the positively charged electrode, where we have seen in Figure 5 that PEO is located in the innermost layer followed by a second layer of TFSI^–^ anions. Probably, the Coulomb attraction between this second anionic layer and the positive electrode is squeezing the polymer against the surface, forcing it to adopt a parallel stacking behavior. However, in the proximity of the negative graphene-like surface, where the polymer was observed to be less constrained in the *z* direction, it does not show a preferential orientation in ternary mixtures with [EMIM][TFSI]-based electrolytes because it has more conformational freedom. Neutral graphene-like surfaces also favor a “flat” polymer conformation, in agreement with previous observations for uncharged Li-metal surfaces.^62,64^ In addition, the amount of IL and the nature of the IL cation do not seem to affect the orientation of PEO molecules. Figure S3 of the Supporting Information shows the orientation of the IL cations that are located in the innermost layer. The results suggest that both IL cations have random arrangements relative to a positively charged electrode, whereas cation rings become more parallel to the negatively charged surfaces in order to get a more efficient accumulation that allows them to overcompensate the charge of the electrode. On the other hand, the orientation of IL cations when adsorbed at neutral surfaces depends on their nature, with imidazolium rings preferring to be parallel regardless of the concentration and pyrrolidinium rings showing no preferential orientation.

**Figure 9:**
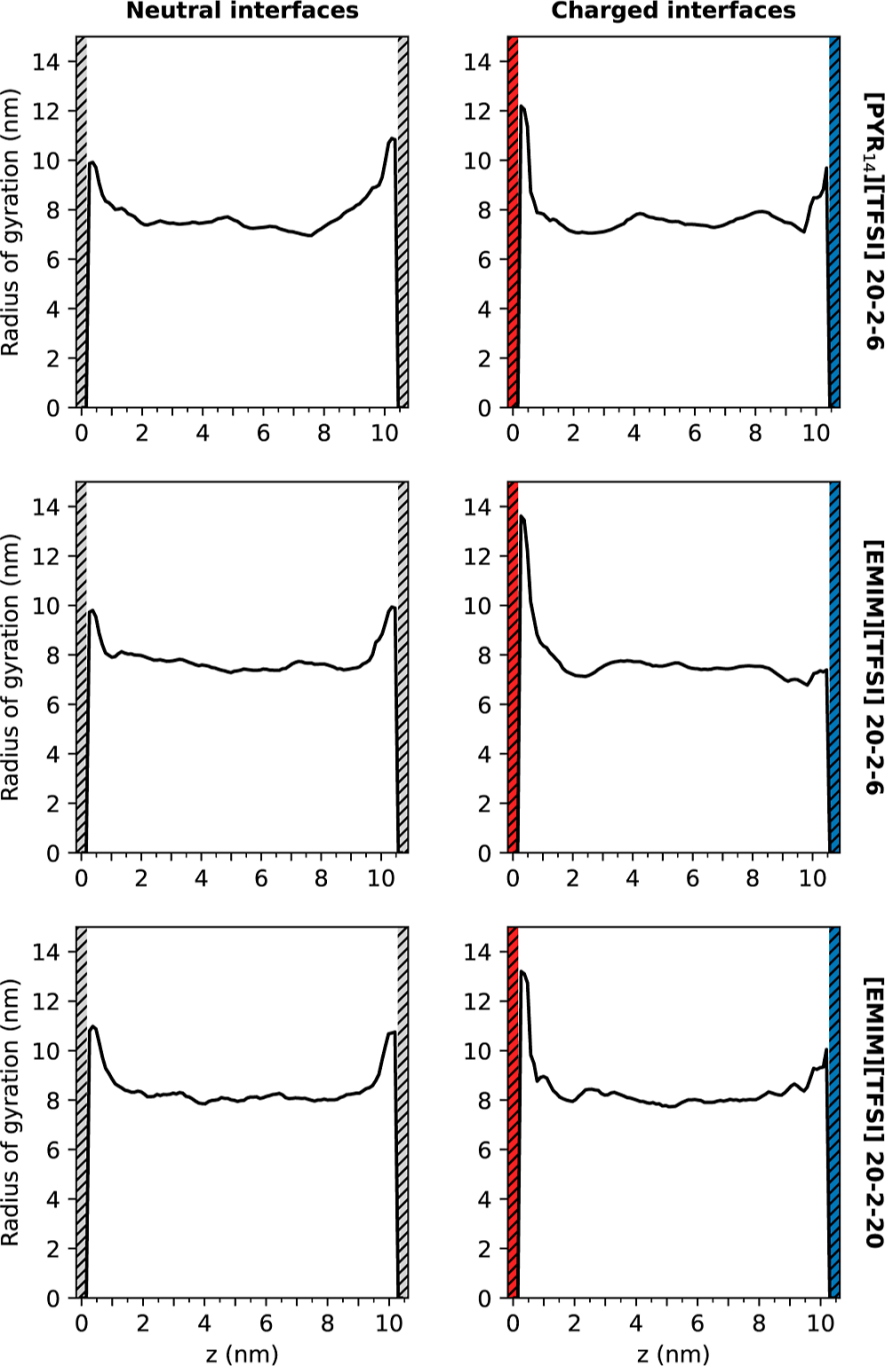
Radius of gyration in the *z* direction for TSPEs confined between neutral (left) and charged (right) graphene-like surfaces.

The interaction of PEO with the components of the solvent and the graphene-like surfaces can be further analyzed by calculating the radius of gyration (*R*_g_) and the end-to-end distance (*d*_e_), which quantify the chain expansion in the mixture and can be calculated, respectively, as
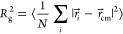
2and
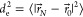
3where *N* is the number of atoms in a PEO chain,  and  are the coordinates of the last and the first atoms of the chain, respectively, and *r*_cm_ is the center of mass of a chain. The results for the radius of gyration as a function of the position of the polymer along the simulation box axis can be seen in Figure 9, whereas those for the end-to-end distance are included in Figure S4 of the Supporting Information. In order to obtain these plots, the computed quantities for each chain were assigned to the *z* coordinate of all of the backbone atoms of the said chain (oxygens and carbons). Our findings show that the radius of gyration increases in the proximity of the neutral and the positively charged walls, which indicates that the PEO backbone is less folded in the interfacial region. This behavior seems to be in open contradiction with the fact that the O(PEO)–Li^+^ interactions were observed to increase close to the graphene-like walls, and the radius of gyration is expected to decrease when increasing those interactions, as previously reported for PEO-based electrolytes confined between Li-metal surfaces.^62,64^ However, as previously stated in this work, PEO molecules in the interfacial region strongly interact with the carbon atoms of the wall, which leads to the unfolding of the polymer. In addition, the radius of gyration is also observed to increase close to the negatively charged surface in mixtures with [PYR_14_][TFSI] due to the enhanced presence of lithium cations and polymer molecules in that interface. In general, the interaction with Li^+^ in the bulk leads to a globule conformation of the polymer on a local scale. However, globally, the constraint introduced by the graphene-like wall and the fact that PEO tries to maximize the interaction with both the surface and all the Li^+^ cations in the interfacial region lead to a transition to a coil conformation. In addition, we computed the gyration tensor in order to further confirm this picture. The gyration tensor of a set of *N* points can be defined as
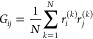
4provided that the center of mass of the points is set to zero. The eigenvalues and eigenvectors of this tensor, or more specifically, the square root of these eigenvalues, offer a measure of the shape of the point cloud. In our case, we can see plotted in Figure S5 the values corresponding to the *Z* direction as well as those corresponding to directions contained in the *XY* plane. These values were computed for all polymers and discretized into bins so that we could see the variation of these parameters along the *Z* axis. As we can see, throughout the simulation box, the square root of the eigenvalues of the *XY* plane is comparable and substantially larger than that of *Z*. This once again confirms our view, suggesting that polymer conformations are disk-shaped and parallel to the surface. This situation is, as we hinted earlier, even more marked at the vicinity of the interface as the value corresponding to the *Z* direction decreases even further in general.

As is well-known, Li^+^ diffusion plays a crucial role in the kinetic process that takes place in battery electrodes and thus in the performance of energy storage devices. To obtain information on Li^+^ dynamics, we analyzed the Li^+^-cation diffusion by calculating the variance Var[Δ*z*] of the displacement vector Δ*z* of these ions as a function of their initial position in *z*, as described in ref (63). The variance of the lithium displacements is calculated as
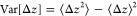
5with ⟨Δ*z*^2^⟩ being the mean-square displacement, and ⟨Δ*z*⟩^2^ the square of the mean displacement in the *z* direction. The brackets indicate the average over all of the particles of the same species and the average over multiple starting times, *t*_*k*_. Thus, the displacement of a given molecule after a lag-time Δ*t* is
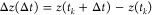
6

**Figure 10:**
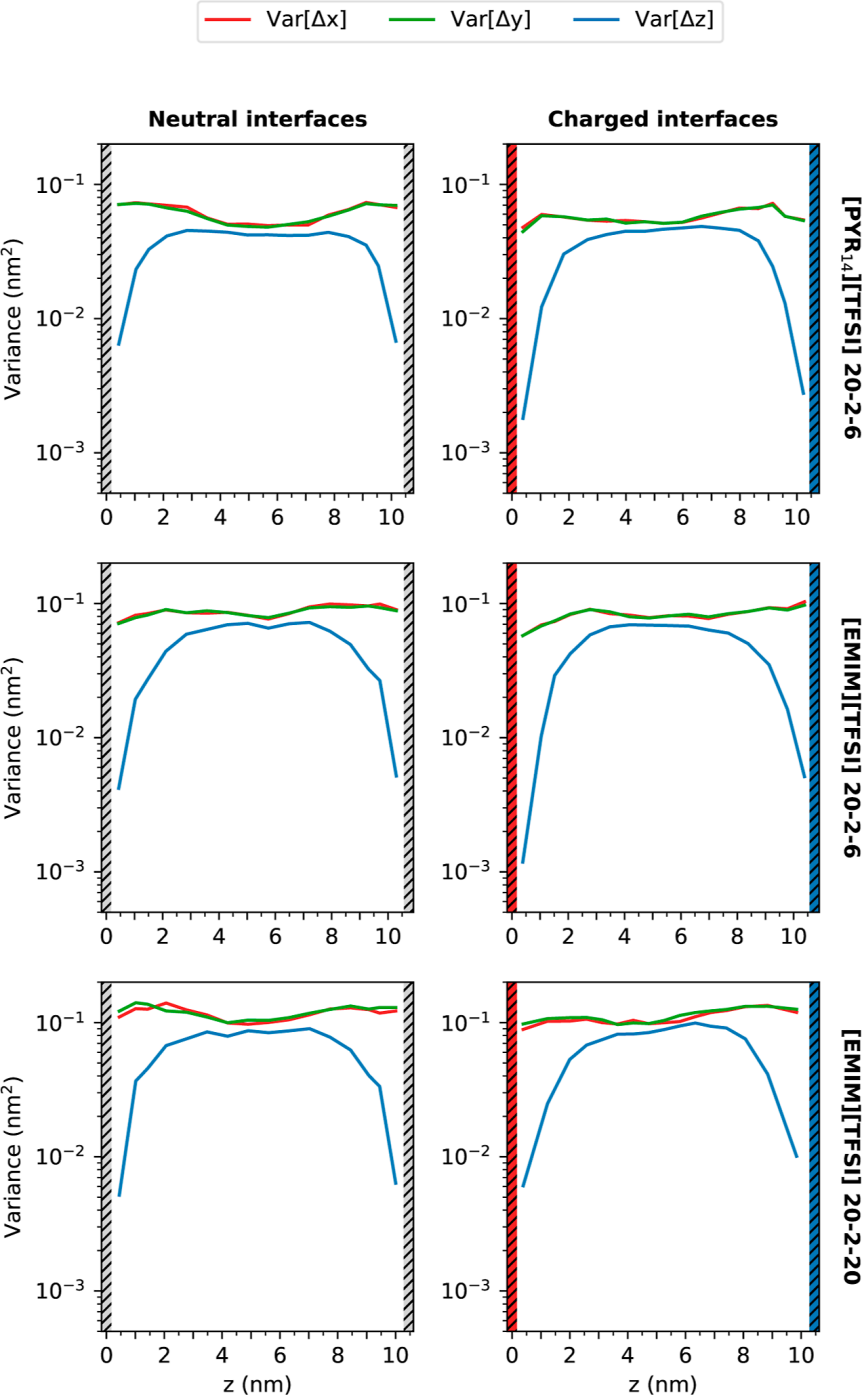
Displacement variance (eq 5) of Li^+^ cations in the three directions as a function of the initial position in *z* for TSPEs confined between neutral (left) and charged (right) graphene-like surfaces.

The variance of the displacements in the *x* and *y* directions were calculated in the same way and all of them are represented in Figure 10 for a lag-time of 1 ns as a function of the initial position in *z*.

It can be observed that, regardless of the charge of the electrodes, of the IL cation, and of the amount of IL, the diffusion of Li^+^ ions in the direction perpendicular to the graphene-like surface always slows in the interfacial region, where the formation of layers takes place. At neutral interfaces, the *z* displacement variance decreases in a similar way for all the systems compared to the bulk, which is consistent with the nearly identical free-energy barriers in the neighborhood of these surfaces, as shown in Figure 7. Charging the electrodes leads to diverse modifications of the *z* displacement variance in the interfacial region for different systems. In general, the shortest *z* displacement variances correspond to those electrolytes with the most stable minima in the free-energy profiles, which is in agreement with Li^+^ cations remaining at the interfaces once they reach that region. These results are indicative of a high interfacial resistance to ionic mobility at both the positively and negatively charged electrode interfaces due to strong interfacial layering. In addition, it can be seen that the bulk behavior is not 100% recovered in the center of the simulation box, since the displacement variances in the direction perpendicular to the electrodes are always lower than those in the *x* and *y* directions.

In the directions parallel to the electrodes, the variance of the displacement shows a much lower degree of dependence with the distance to the carbon interface compared to the perpendicular direction. In addition, Li^+^-cation diffusion is slightly faster in the regions of the outer layers of the electric double layer (EDL) and then decreases again in the inner layers, probably due to a constraining effect due to the presence of other species or to the own charge of the electrode. In summary, the molecular conformation at the innermost layer of the EDL inhibits Li^+^ transport in the three directions, but their migration toward the electrode suffers a much more marked “locked-in-place” effect once within the interfacial layering.

From the analysis of the displacement variance, we can also infer that Li^+^ mobility slightly increases with increasing IL concentration, both between neutral and charged graphene-like surfaces, which can be indicative of the IL acting as a plasticizer. Also, the comparison between both ILs at a proportion of 20–2–6 shows that the addition of [EMIM][TFSI] leads to moderately enhanced Li^+^ dynamics.

The variance of the displacements of PEO oxygens is included in Figure S6 of the Supporting Information. There it can be seen that PEO diffusion shows, in general, the same features as those of Li^+^ ions. We can observe the same restraint in the motion perpendicular to the electrodes when the oxygen atoms are located close to the interface. It is interesting to notice that the variance in this direction never reaches values comparable to those of the *x* and *y* directions. This effect is likely due to the much larger size of the PEO chains compared to the other molecular species. This way, the chains could “feel” the presence of the interfaces even when part of it is far away from the electrode.

### Influence of Adding IL into the Binary SPE (PEO/LiTFSI)

As is well-known, the addition of an IL to an SPE leads to a plasticizing effect that enhances the mobility of the Li^+^ cation, which confers ideal properties to the TSPEs. Understanding the role of the IL in the structure of these ternary mixtures is therefore of paramount importance. In this section, we will attempt to shed some light on this issue. Previously, Thum et al.^63^ reported structural results for an SPE composed of both PEO and [Li][TFSI] in the presence of neutral and charged graphene-like surfaces.

Concerning Li^+^ solvation in bulk, the main effect of IL inclusion is the appearance of some degree of coordination with TFSI oxygen atoms, which was hardly present for PEO–LiTFSI binary mixtures at the expense of decreasing that with PEO oxygens. The total number of 6 oxygens around a given lithium is not changed with the addition of the IL, but the coordination environment of 6 PEO oxygens observed by Thum et al.^63^ is more markedly modified when increasing the amount of [EMIM][TFSI], even being swung up to a solvation shell composed of 4 and 2 oxygens from TFSI and PEO, respectively, which can be attributed to a dilution of the polymer. In the proximity of graphene-like surfaces, the addition of an IL to the binary SPE affects the surroundings of Li^+^ cations in a less pronounced way compared with the bulk, but the results depend on the charge of the electrode. For neutral surfaces, the insertion of an IL leads to Li^+^ being surrounded not by 5 PEO oxygens but instead by 4 and 2 oxygen atoms from PEO and TFSI, respectively. This proportion is reversed with increasing IL concentration. Concerning the positively charged surface, we observed that the interaction with the polymer decreases from 5 PEO oxygens to 3–4 when including any of the ILs, whereas the number of TFSI oxygens is kept at 2–3. This shows that the charge of the surface dominates over the addition of the IL, as this is the electrode that electrostatically attracts the anions, which increases the interaction between TFSI^–^ and solvated Li^+^. On the other hand, near the negative electrode, the structural picture depends on the concentration of IL. At low amounts of IL, the 2–3 PEO oxygens coordinating a Li^+^ observed in the binary SPE are kept in ternary mixtures, although the inclusion of IL leads to the incorporation of two TFSI oxygens to the Li^+^ first solvation shell. At high [EMIM][TFSI] concentrations, the anions take 4–5 of the 6 interaction sites of Li^+^.

The comparison of the number density profiles with those reported by Thum et al.^63^ reveals that, near neutral graphene-like surfaces, the addition of IL does not markedly modify the composition of the innermost layer of the EDL, which is filled up with anions and polymer molecules. Also, the peak positions of Li^+^ do not change relative to those observed by Thum et al.^63^ However, while they obtained a similar height for both peaks, in TSPEs, there is a clear tendency of Li^+^ ions to be placed as close as possible to the electrode. On the other hand, the layer that is in direct contact with the positively charged surface is still shared by polymers and anions regardless of the inclusion of IL. Notwithstanding, for low IL concentrations, a new position of Li^+^ appears at around 0.6 nm, which probably corresponds to those lithiums that are dragged by the anions. With respect to the negative electrode, some noteworthy changes are observed when adding IL to the binary SPE studied by Thum et al.^63^ First, the positions close to the surface at which PEO molecules are located in binary mixtures are now occupied by the IL cations in the ternary ones. Second, this leads to a competition between Li^+^ and IL cations to reach the negatively charged surface, which is more remarkable when increasing the amount of IL, as expected. In fact, at the highest [EMIM][TFSI] concentration, the negative electrode is observed to be fully impregnated by EMIM^+^ and Li^+^ and neither PEO is able to get to the innermost layer.

The number density profiles are directly related to the energy landscape that Li^+^ has to cross to reach the electrodes. Those free-energy profiles are much smoother with the addition of IL, in particular, in those systems with neutral graphene-like walls, where the energetic barrier almost vanishes. Previous results reported by Thum et al.^63^ for neutral surfaces showed a much more layered profile for the binary SPE, where lithium cations need to overcome several barriers before reaching the interfacial region. In the absence of Coulomb interactions with the electrodes that add competing effects into the systems, these smoother energy profiles can be indicative of a reduced degree of ordering with the addition of IL that facilitates the motion of Li^+^ through the simulation box toward the surfaces. Although we cannot establish a clear connection between the well-known plasticizer effect of the IL from our MD simulations and those of Thum et al.,^63^ the comparison between the free-energy profiles at charged interfaces indicates that, for choosing the amount of IL to be added to the binary SPE, a compromise between enhancing PEO and Li^+^ motion and how the IL ions are going to compete for the electrodes must be made. In this regard, our results advise us to avoid very high IL concentrations.

## Conclusions

In the present work, classical MD simulations were performed for TSPEs [containing the polymer PEO, a lithium salt ([Li][TFSI]), and an IL] confined between two graphene-like surfaces. Two ILs at different concentrations were tested, [PYR_14_][TFSI] and [EMIM][TFSI], with the aim of determining the optimal composition of the electrolyte system for application in energy storage devices. The structural picture of the molecular species in the interfacial region with both charged and uncharged walls was characterized by the RDFs, the coordination numbers, the number density profiles, and the orientations. The dynamics and kinetics of Li^+^ motion were analyzed in terms of the variance of the displacement and the free-energy profiles. In addition, the PEO conformation was investigated through calculation of the radius of gyration and the end-to-end distance.

All systems show the typical electrolyte–electrode interfacial layering characteristic. This results in Li^+^ being preferentially coordinated with the PEO oxygens instead of with TFSI^–^ anions in the neighborhood of neutral graphene-like surfaces, which is the opposite behavior of the one previously observed for Li-metal electrodes. The innermost layer in the neutral interfacial region is mainly composed of PEO molecules, which are arranged parallel to the surface in a “flat” conformation trying to maximize their interaction with the hydrophobic graphene. In the presence of charged graphene-like surfaces, lithium cations more easily approach the negatively charged electrode with the pyrrolidinium-based TSPE, rather than with the imidazolium-based one even for high concentrations, which is probably due to the lower degree of anion clustering with Li^+^ in the former case. This allows lithium cations to coordinate with the polymer and reach the negative surface. However, the analysis of the free-energy profiles shows that the barriers lithium cations must overcome to come into contact with the negative electrode are much higher than the thermal energy, so the diffusion process can be regarded as a rare event, which, in the case of taking place, would lead to very stable Li^+^ adsorption. Concerning the dynamics, the diffusion of Li^+^ ions in the direction perpendicular to the graphene-like surfaces always slows down regardless of their charge due to the strong interfacial layering that leads to a high interfacial resistance to ionic mobility.

In summary, although our findings indicate that large amounts of imidazolium-based ILs increase Li^+^ mobility due to a plasticizing effect, we also observed that the presence of the [PYR_14_^+^] cation energetically favors Li^+^ presence at the negative electrode when compared to [EMIM^+^]. Thus, we suggest the use of low amounts of pyrrolidinium-based ILs as the proper selection of the TSPE in order to optimize solid-like battery performance.

## Supporting Information Available

RDFs of binary mixtures IL + LiTFSI, orientations of both IL cations, and displacement variance of PEO oxygens (PDF)
