# Supplementary material for: Ternary Solid Polymer Electrolytes at the Electrochemical Interface: A Computational Study
Source: Macromolecules. 2024 Apr 29;57(9):3921–36. doi: 10.1021/acs.macromol.3c02669 (PMC11100289; doi:10.1021/acs.macromol.3c02669)
Supplement: Supplementary file 1 — ma3c02669_si_001.pdf [file ma3c02669_si_001.pdf]

Supporting Information: Ternary solid polymer electrolytes at the electrochemical interface: a computational study

Alejandro Rivera-Pousa,[†,‡] José Manuel Otero-Mato,[†,‡] Hadrián Montes-Campos,[*,†,‡,¶] Trinidad Méndez-Morales,[†,‡] Diddo Diddens,[§] Andreas Heuer,[‖,§] and Luis Miguel Varela[*,†,‡]

†*Grupo de Nanomateriais, Fotónica e Materia Branda, Departamento de Física de Partículas, Universidade de Santiago de Compostela, Campus Vida s/n, E-15782, Santiago de Compostela, Spain*

‡*Instituto de Materiais (iMATUS), Universidade de Santiago de Compostela, Avenida do Mestre Mateo 25, E-15782, Santiago de Compostela, Spain*

¶*CIQUP, Institute of Molecular Sciences (IMS)—Departamento de Química e Bioquímica, Faculdade de Ciências da Universidade do Porto, Rua Campo Alegre, 4169-007, Porto, Portugal*

§*Helmholtz-Institute Münster (HI MS), Ionics in Energy Storage, Forschungszentrum Jülich GmbH, Corrensstraße 46, 48149 Münster, Germany*

‖*Institute of Physical Chemistry, University of Münster, Corrensstraße 28/30, 48149, Münster, Germany*

E-mail: hadrian.montes@usc.es; luismiguel.varela@usc.es

Results and Discussion

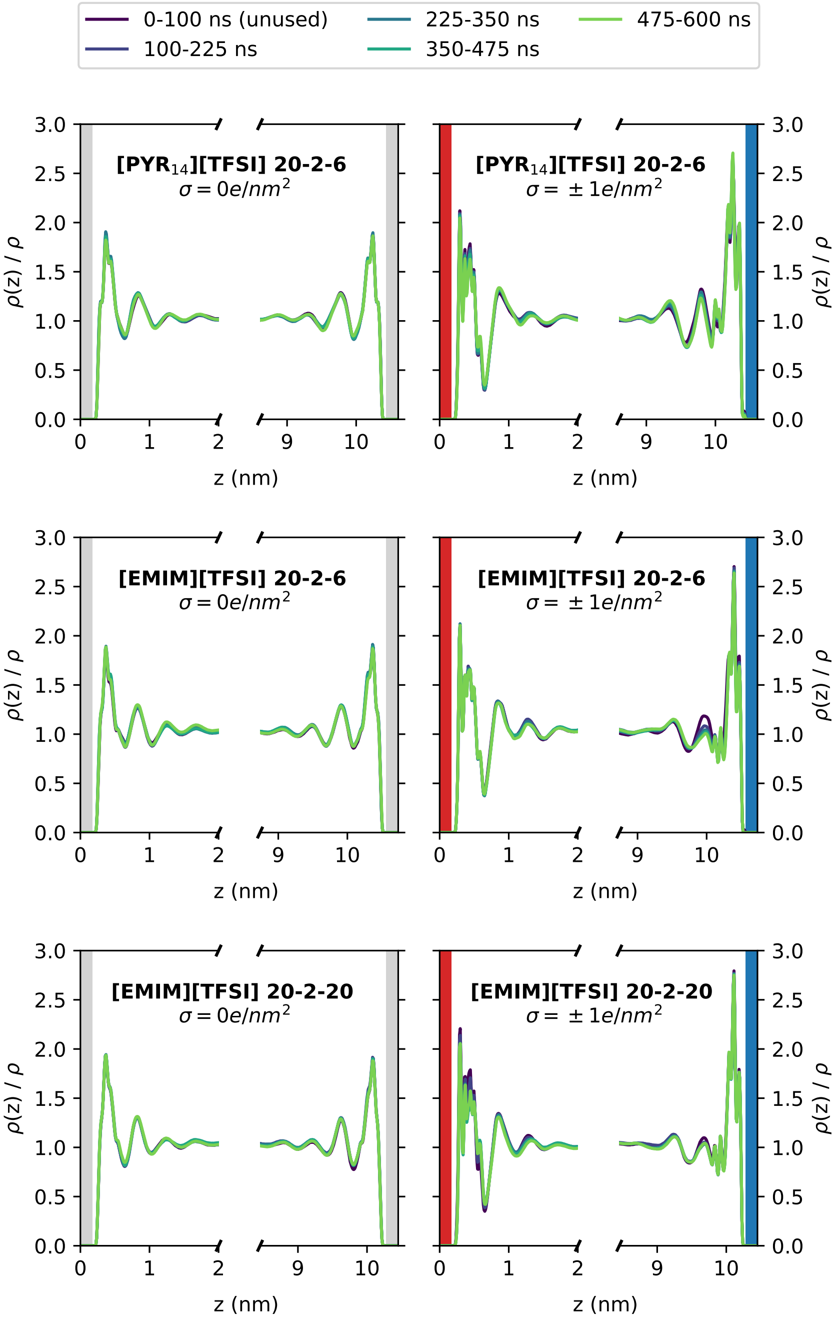

Figure S1: Evolution of the density profiles along the Z axis of the whole simulation system. It can be seen that for the first 100 ns the system is not well stabilized yet, and thus that interval time is discarded from the production run.

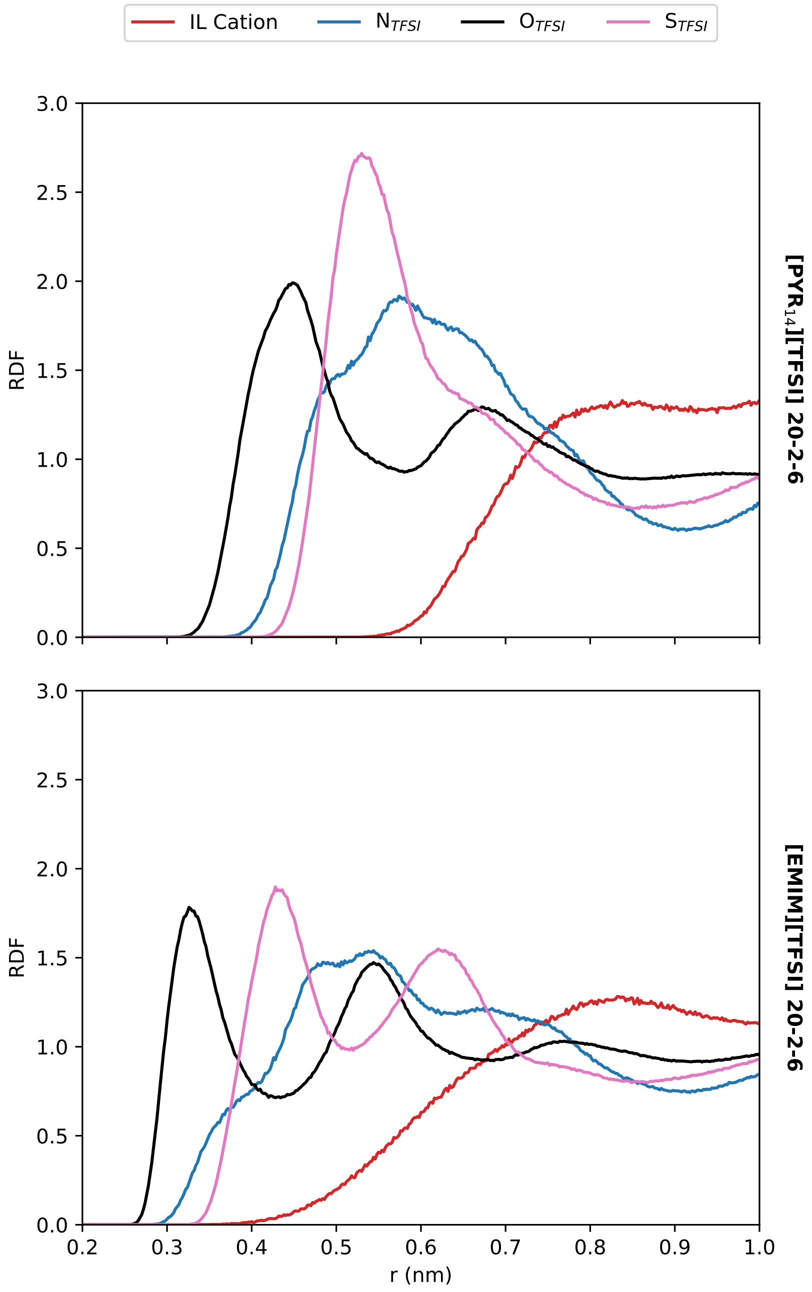

Figure S2: Radial distribution functions between (top) $[Pyr_{14}]^+$ cation and $TFSI^-$ anion and (bottom) $[EMIM]^+$ cation and $TFSI^-$ anion, both in binary mixtures with LiTFSI. The ratio salt/IL is 2-6, which corresponds to the lowest IL concentration in ternary SPEs.

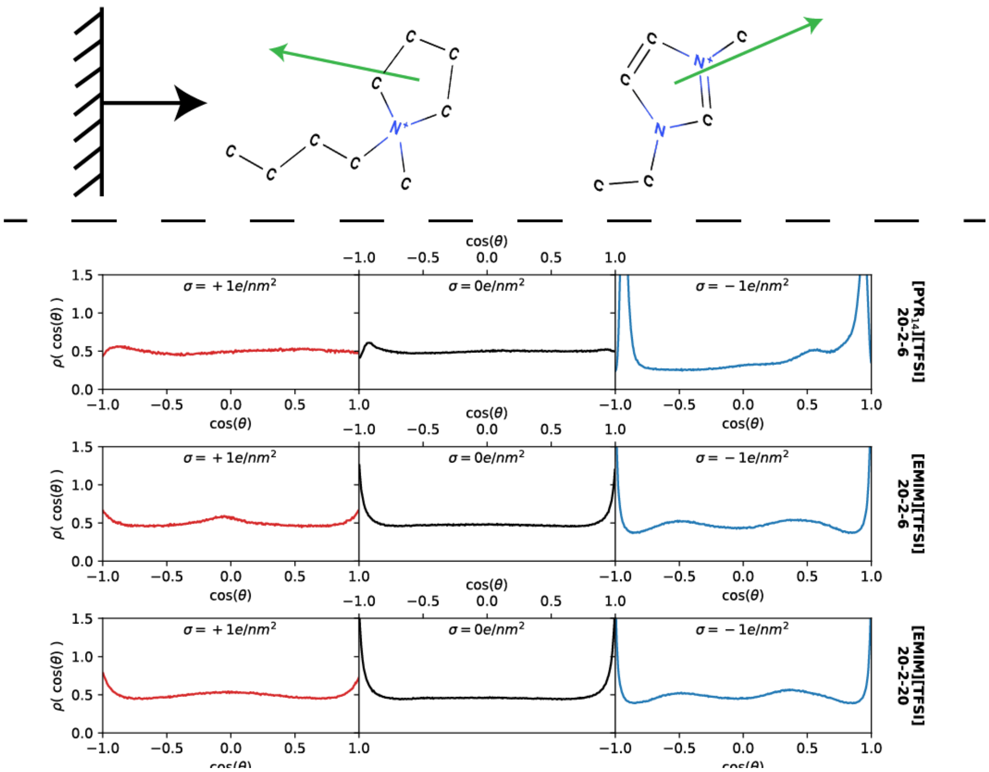

Figure S3: Marginal probability density distribution of IL cation orientation with respect to a vector normal to the graphene-like surface and pointing to the bulk, in the interfacial region (up to 2 nm). The characteristic vectors of the cations are schematized on top of the figure.

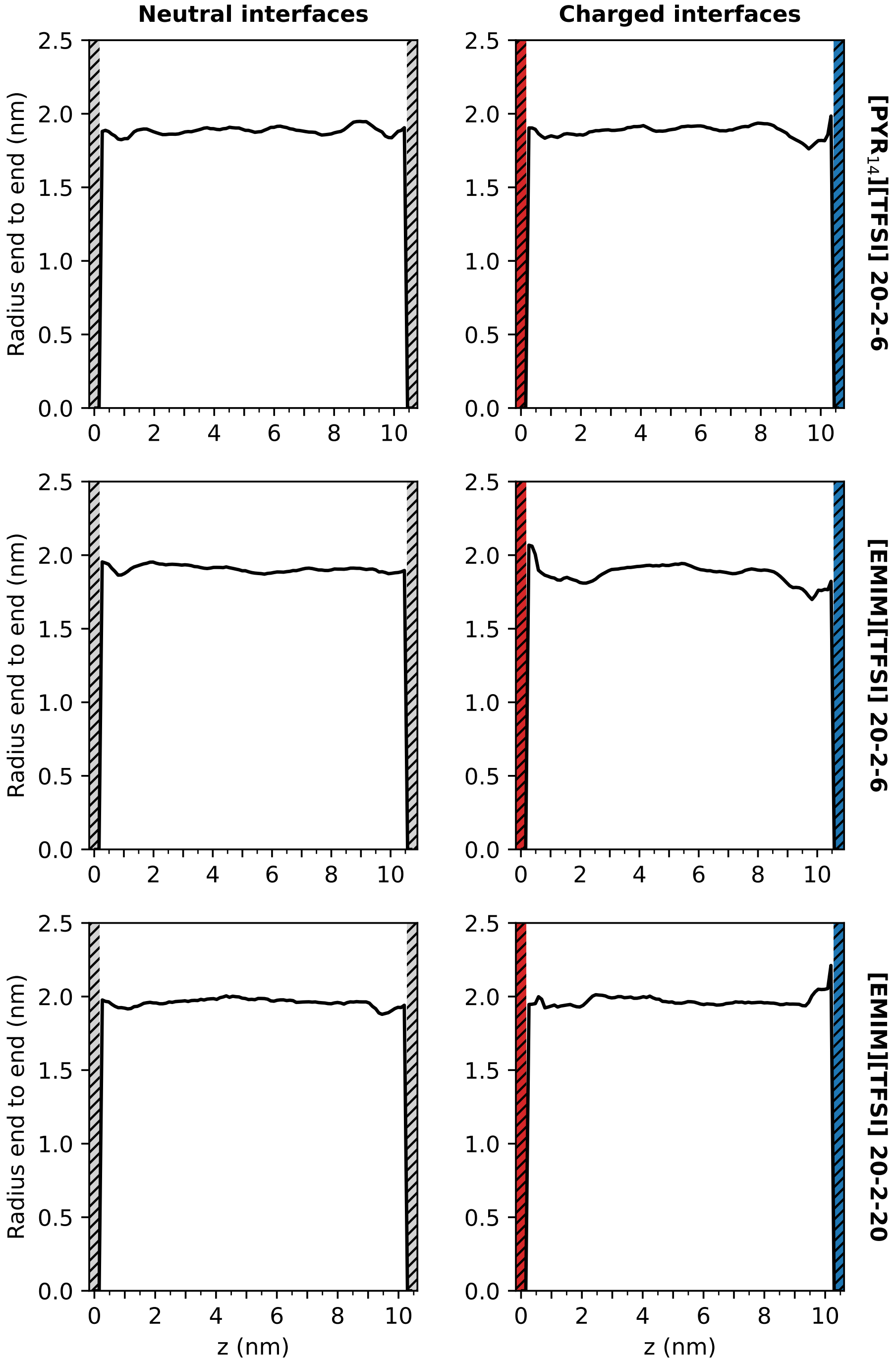

Figure S4: End-to-end distance in the z direction for TSPEs confined between neutral (left) and charged (right) graphene-like surfaces.

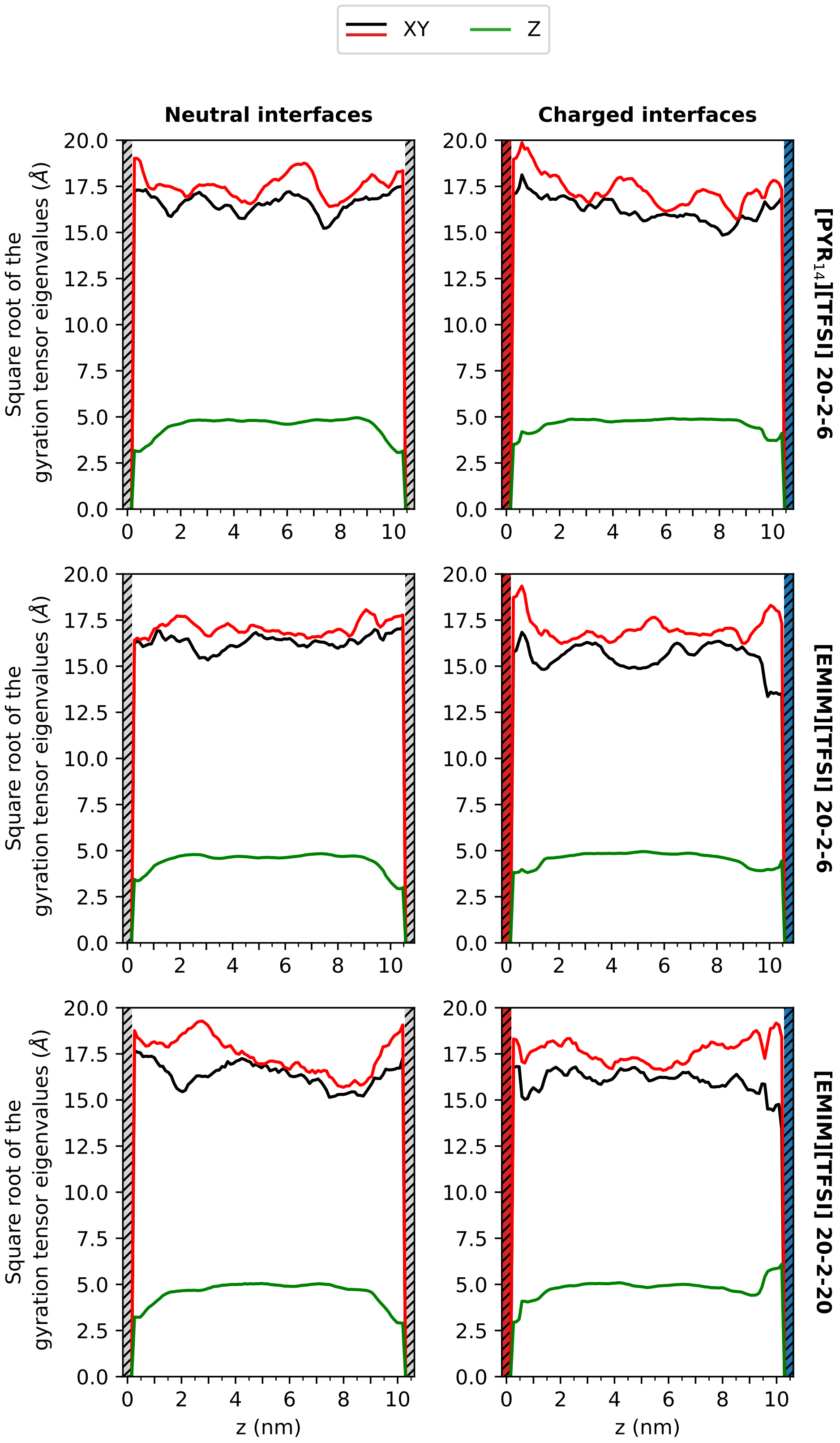

Figure S5: Square root of the eigenvalues of the gyration tensor corresponding to the Z direction (green) and directions contained in the XY plane (red and black) for TSPEs confined between neutral (left) and charged (right) graphene-like surfaces.

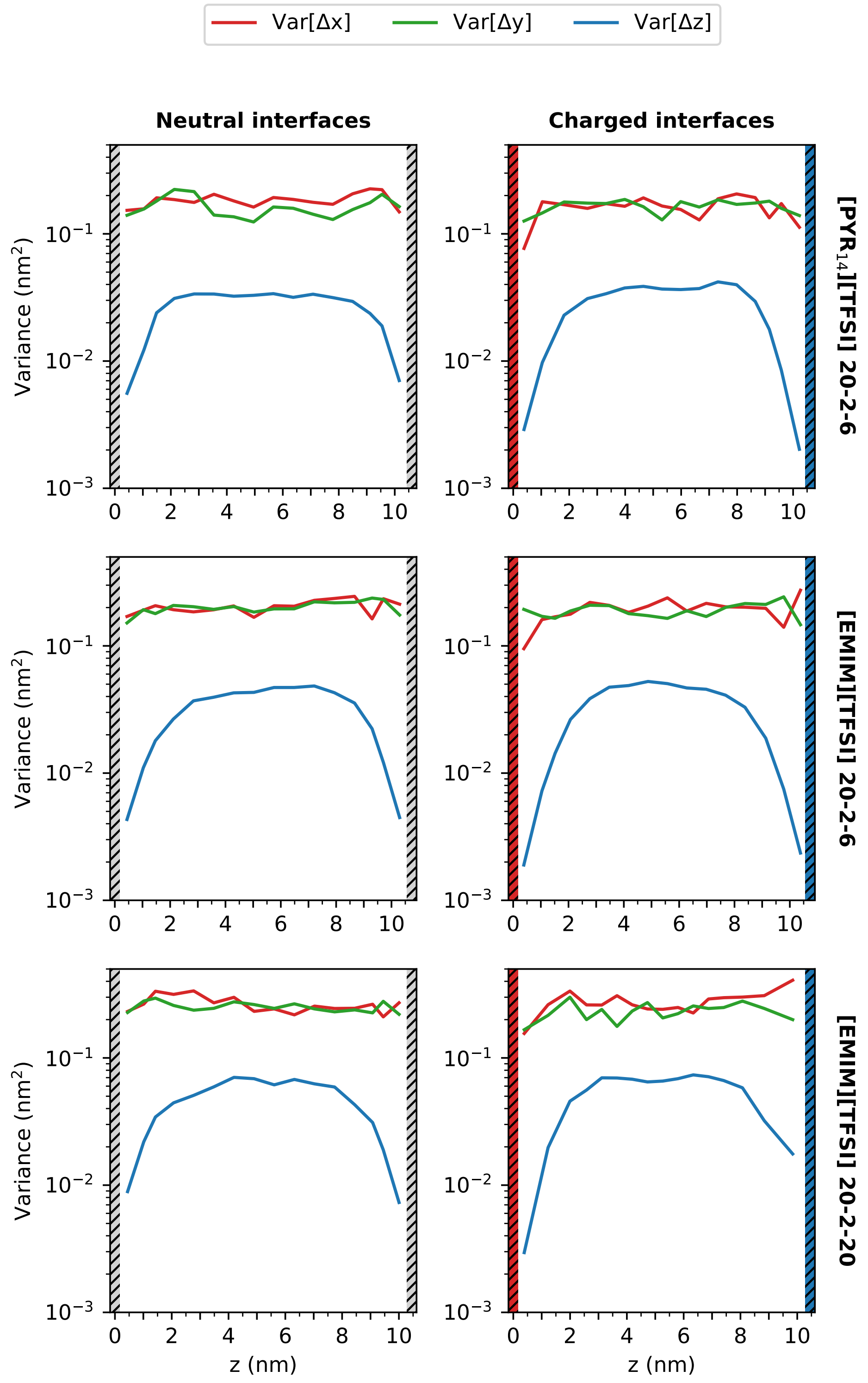

Figure S6: Displacement variance of PEO oxygens in the three directions as a function of the initial position in z for TSPEs confined between neutral (left) and charged (right) graphene-like surfaces.
